# Barriers to Physical Activity in Spanish Children and Adolescents: Sex and Educational Stage Differences

**DOI:** 10.3389/fpsyg.2022.910930

**Published:** 2022-06-03

**Authors:** José Manuel Delfa-De-La-Morena, Daniel Bores-García, Adrián Solera-Alfonso, Nuria Romero-Parra

**Affiliations:** ^1^Department of Physiotherapy, Occupational Therapy, Physical Medicine and Rehabilitation, Rey Juan Carlos University, Móstoles, Spain; ^2^Research Group of Humanities and Qualitative Research in Health Science, Rey Juan Carlos University, Madrid, Spain; ^3^LFE Research Group, Department of Health and Human Performance, Faculty of Physical Activity and Sports Science, Universidad Politécnica de Madrid, Madrid, Spain

**Keywords:** MVPA, exercise, inhibitors, high-school, kids

## Abstract

According to worrisome childhood obesity and inadequate physical activity (PA) levels worldwide, especially exacerbated in adolescents girls, this work aimed to identify sex and educational stage differences in barriers to meet PA requirements and international guidelines in Spanish children and adolescents considering the entire educational pathway (primary, secondary, and college). The Short Form of the International PA Questionnaire and the Scale of Perceived Barriers were administered to primary, secondary, and college education students (13,491 boys and 13,238 girls, 9–19 yrs). Two-way ANOVA was performed to analyze barriers to PA according to sex and educational stage with physical status as covariate. Higher disliking and time barriers were reported by females (1.5 ± 1.2 and 3.2 ± 1.5 points) in comparison to males (1.2 ± 1.0 and 2.8 ± 1.4), while primary students showed lower disliking and time (1.2 ± 1.0 and 2.8 ± 1.5) and higher safety (3.1 ± 1.8) constraints in comparison to secondary (1.4 ± 1.1, 3.1 ± 1.4, and 2.8 ± 1.7) and college (1.5 ± 1.2, 3.2 ± 1.5, and 2.8 ± 1.6; *p* < 0.05 for all comparisons). College females showed higher disliking (1.7 ± 1.2) and time (3.5 ± 1.4) barriers than secondary females (1.5 ± 1.2 and 3.3 ± 1.4; *p* < 0.05). Sex and educational stage were determinant for time and dislike of PA barriers, which were rated higher by female students in comparison to their male counterparts and from primary education onwards. Altogether this, suggests promotion strategies should carefully consider girls and the step into secondary school.

## Introduction

A growing body of evidence shows the benefits of regular physical activity (PA) (Poitras et al., 2016; Biddle et al., 2019) and the negative effects of sedentary lifestyles. In fact, PA abandonment at an early age have potential repercussions throughout the lifespan and even shorten the years of life (Pinheiro Gordia et al., 2015). Unfortunately, inadequate levels of PA have been observed among adolescents (Kurdaningsih et al., 2016). There are currently more than fifty national and international guidelines on the type, intensity and frequency of PA recommended for each age group (Parrish et al., 2020), being those of the World Health Organization (WHO) the best known and globally recognized (Bull et al., 2020). According to these recommendations adolescents should engage in at least 60 min of mostly aerobic PA of moderate to vigorous intensity per day (MVPA), in addition to strength training 3 days per week. However, the reality is that more than 77% of boys and 84% of girls in adolescence worldwide show insufficient levels of PA in relation to these international guidelines (Palou et al., 2019; Guthold et al., 2020) with higher percentages in populations belonging to lower socioeconomic strata (Martins et al., 2021).

This trend, which has been growing in recent decades, has generated a serious public health problem due to the increase in diseases and problems related to overweight and obesity (Vasquez et al., 2021). Spain is one of the countries in the world with the highest rates of childhood overweight (20.7%) and obesity (14.2%) with an increase of two percentage points in the last two decades (Gómez et al., 2020; Mendoza-Muñoz et al., 2020). The factors that most hinder the practice of regular PA are the lack of time (Calogiuri and Chroni, 2014; Chacón-Cuberos et al., 2017; Jodkowska et al., 2017; Rodrigues et al., 2019; Alves et al., 2021), economic cost (Rodrigues et al., 2019), overuse of technologies (Divyasree et al., 2018; Harvey et al., 2018), lack of energy and willpower (Jodkowska et al., 2015, 2017; Rosselli et al., 2020), lack of family and institutional support (Stanley et al., 2012; Alsubaie and Omer, 2015; Vasquez et al., 2021), or shortage of accessible sports facilities (Alsubaie and Omer, 2015; Divyasree et al., 2018; Marconnot et al., 2019).

Regarding sex and age differences, girls seem to be more prone to inactivity than boys, reporting a gender imbalance in the sports offer and in the policies for the promotion of physical activity, more oriented to the male population (Madsen et al., 2009; Guthold et al., 2020; Rosselli et al., 2020). In addition, a gradual decline in PA levels has been observed in the transition from primary school to adolescence, mainly due to changing priorities in the use of leisure time, increased academic demands and a lack of motivation to engage in physical activity when the social relationships involved are not sufficiently satisfactory (Dumith et al., 2011; Jago et al., 2012; Van Hecke et al., 2016). This pattern of higher levels of PA practice in boys has also been observed in Spanish adolescents (Sánchez-Miguel et al., 2017), being the most decisive barriers to PA practice the lack of time, the participation in leisure activities to which they give a privileged position with respect to PA and the lack of motivation due to the imposition of unchosen activities. Additionally, girls reported concerns about body image and diet in relation to PA (Fernández-Prieto et al., 2020). However, after a thorough review of the literature, no studies in the Spanish population have researched on these sex and age differences in barriers to PA with representative samples of children and adolescents from different grades of the educational system (primary to college). We hypothesized that female students rate higher the barriers to meet PA levels, especially the time-related ones, than males, being these barriers accentuated from the adolescence period. Therefore, the purpose of the present study was to identify sex and educational stage differences in the existing barriers to PA in children and adolescents from 5th grade of primary school to 2nd grade of college.

## Methods

### Participants and Study Design

Participants from all stages within the entire educational pathway (primary, secondary, and college) were recruited from different schools of Madrid Region to participate in this cross-sectional study. Madrid Region is located in the center of Spain and is the 3rd most populated region of the country out of 17 regions, with 6.8 million inhabitants, being the total population of Spain 47.4 million inhabitants. However, within the region, a wide range of population levels is present, which makes the sample representative of either overpopulated urbanized areas or underpopulated rural areas commonly found in Spain. Inhabitants' information from the different areas of Madrid where data were collected are presented in Table 1. Levels of MVPA and barriers to PA were examined in a total of 26,729 students, with a balanced distribution by sex (13,491 boys and 13,238 girls), from 5th and 6th levels of primary school (*n* = 11,122, 11.7 ± 1.9 years), 1st, 2nd, 3rd, and 4th levels of secondary school (*n* = 12,379, 14.5 ± 2.0 years) and 1st and 2nd levels of college (*n* = 3,228, 17.6 ± 2.3 years). Participants were classified as non-active (*n* = 17,803) or active (*n* = 8,926) considering the accomplishment of 60 min per day of MVPA according to WHO guidelines (Bull et al., 2020). An informative introductory letter was sent to all schools of Madrid Region and those volunteered to accept participated in the study. Participants and their parents when appropriate were asked to read and sign a consent form. All procedures complied with the Declaration of Helsinki and were approved by the Universidad Rey Juan Carlos ethics committee board (registration number 1306201809818).

**Table 1:** Inhabitants per area in Madrid Region.

	**Inhabitants**	
**Area**	***N***	**Mean ±SD**
City center	6,315	166386.5 ± 53901.4
North	2,732	76437.4 ± 32708.3
South	6,165	145462.9 ± 64767.0
East	2,400	109453.7 ± 67232.2
West	3,595	54566.2 ± 26091.9
Nordwest	304	7762.2 ± 4856.6
Southwest	1,699	14873.4 ± 12031.8
Southeast	972	34255.3 ± 38409.7
Central M.	1,643	10966.3 ± 4575.9
Northern M.	610	3543.2 ± 1957.0
Southern M.	294	3223.8 ± 2103.3
Avg	26,729	100910.7 ± 75509.1

*Avg, average; M, mountains*.

### Instruments

The International Physical Activity Questionnaire Short-Form (IPAQ-SF) was used to evaluate PA levels (Craig et al., 2003). The instrument was developed to provide cross-national information of PA, and this short form showed acceptable reliability and validity getting more acceptance between both investigators and respondents (Craig et al., 2003). It, has been previously administered in children and adolescents (Pandolfo et al., 2016; Brand et al., 2017; Duncan et al., 2017; Sánchez-Miguel et al., 2017) and it has been validated in Spanish population (Roman-Viñas et al., 2010; Román Viñas et al., 2013).

To assess perceived barriers the instrument used was the Scale of Perceived Barriers (Chinn et al., 1999) which has been previously used in Spanish population (Zaragoza et al., 2011). Prior to completing the questionnaire, participants were provided with the definition of barriers toward PA as factors that may prevent an individual from being physically active. The questionnaire consisted of 17 items preceded by the sentence “*How much of a problem are the following reasons for you to do physical activity?”*. Each item's response was graded on a Likert scale from 0 being “no problem at all to perform PA” (and hence no barrier was considered), to 6 meaning “a reason that is very likely to prevent PA from being performed”. The 17 items were grouped into four categories or constructs whose internal reliability was previously assessed (Cronbach's Alpha values ≥ 1.0 were retained and a factor loading cutoff of 0.45 were considered to be significant; Zaragoza et al., 2011): Disliking physical activity with 8 items (e.g., “*Not good at physical activity and sports”)*, Time constraints with 4 items (e.g., “*I have too much school work”)*, Safety reasons with 2 items (e.g., “*Physical activity outdoors is not safe”*) and Environmental/contextual reasons with 3 items (e.g., “*I don't have the right equipment”*).

### Statistical Analysis

Data are presented as mean ± SD. The statistical analysis was conducted using the software package SPSS for Windows, version 27.0 (IBM Corp, Armonk, NY). A Kolmogorov-Smirnov test for normality was used. Two-way ANOVA (sex × stage) was performed to examine MVPA. A *t*-test was performed to compare differences in barriers to PA between non-active and active participants. Two-way ANOVA (sex × stage) was performed to analyze barriers toward PA practice but using individuals non-active or active status as covariate for environmental and disliking domains since differences between these two groups were observed from the *t*-test. Where appropriate, the Bonferroni *post-hoc* test was applied to examine pairwise comparisons of each significant factor. The ES was calculated by partial eta-squared ($\eta_{p}^{2}$) which was interpreted based on the following: small, moderate, and large effect for values greater than 0.010, 0.059, and 0.138, respectively (Cohen, 2013). The alpha level was set at *p* < 0.05.

## Results

Effects of sex [*F*_(1, 26)_ = 634.14, *p* < 0.001, $\eta_{p}^{2}$ = 0.023], educational stage [*F*_(2, 26)_ = 28.10, *p* < 0.017, $\eta_{p}^{2}$ = 0.002] and interaction [*F*_(2, 26)_ = 28.10, *p* < 0.032, $\eta_{p}^{2}$ = 0.001] between both factors were observed for MVPA levels, indicating that boys perform more min of PA (58.6 ± 45.9 min) than girls (43.4 ± 38.7 min), in each educational stage (primary: 60.1 ± 47.0 and 46.4 ± 40.0; secondary: 57.8 ± 44.9 and 41.7 ± 33.6; college: 56.7 ± 45.9 and 39.6 ± 37.7 min, respectively, for males and females) being the primary school the stage with more active students (53.3 ± 44.2 min) in comparison to secondary (49.9 ± 42.3 min) and college (48.0 ± 42.8 min).

Barriers results include data from participants indicating the presence of barriers (punctuation > 0 in the questionnaire). Results from *T*-tests revealed higher values for Environmental and Disliking barriers (*t* = 6.80 and *t* = 28.87, respectively, *p* < 0.001 for both comparisons) in non-active (1.7 ± 1.2 and 1.5 ± 1.2 points, respectively) vs. active (1.6 ± 1.2 and 1.0 ± 0.9 points) participants. No differences were observed for Safety and Time domains between non-active (1.7 ± 1.2 and 1.5 ± 1.2 points) and active (1.7 ± 1.2 and 1.5 ± 1.2 points) students (p>0.05). Hence, being non-active or active was used as covariate to explore differences in Environmental and Disliking barriers to PA according to sex and educational stage.

Results and main effects of sex, stage, and interaction between both variables are presented in Table 2. The covariate “non-active vs. active” based on the accomplishment of the 60 min of MVPA resulted significant for both Environmental and Disliking barriers with $\eta_{\text{p}}^{2}$ values 0.003 and 0.025, respectively (*p* < 0.001 for both domains).

**Table 2:** Barriers to PA according to sex and educational stage.

							**Main effects**		
		**Primary**	**Secondary**	**College**	**Avg**		**Sex**	**Stage**	Sex^*^Stage
Environmental	Males	1.65 ± 1.22	1.69 ± 1.20	1.68 ± 1.19	1.67 ± 1.21	*F* =	1.425	0.537	0.093
	Females	1.71 ± 1.25	1.73 ± 1.25	1.72 ± 1.20	1.72 ± 1.24	*p* =	0.233	0.584	0.911
	Avg	1.68 ± 1.23	1.71 ± 1.23	1.70 ± 1.19	1.70 ± 1.23	$\eta_{p}^{2}$ =	0.001	0.001	0.001
Disliking	Males	1.11 ± 0.97^**§**^	1.21 ± 1.04^**§**^, ^*^	1.26 ± 1.03^**§**^, ^*^	1.18 ± 1.01^**#**^	*F* =	227.945	76.114	11.851
	Females	1.31 ± 1.10	1.54 ± 1.19^*^	1.67 ± 1.22^**†**^, ^*^	1.47 ± 1.17	*p* =	**<0.001**	**<0.001**	**<0.001**
	Avg	1.22 ± 1.05	1.38 ± 1.13^*^	1.48 ± 1.15^^*^, †^	1.33 ± 1.10	$\eta_{p}^{2}$ =	0.010	0.007	0.001
Safety	Males	3.03 ± 1.84	2.82 ± 1.75	2.80 ± 1.69	2.91 ± 1.78	*F* =	0.858	24.524	0.035
	Females	3.07 ± 1.81	2.86 ± 1.70	2.82 ± 1.65	2.94 ± 1.73	*p* =	0.354	**<0.001**	0.966
	Avg	3.05 ± 1.83	2.84 ± 1.71^*^	2.80 ± 1.66^*^	2.92 ± 1.76	$\eta_{p}^{2}$ =	0.001	0.004	0.001
Time	Males	2.65 ± 1.43^**§**^	2.91 ± 1.42^**§**^, ^*^	2.94 ± 1.45^**§**^, ^*^	2.81 ± 1.44^**§**^	*F* =	324.550	225.891	18.920
	Females	2.88 ± 1.49	3.33 ± 1.44^*^	3.48 ± 1.43^**†**^, ^*^	3.16 ± 1.48	*p* =	**<0.001**	**<0.001**	**<0.001**
	Avg	2.77 ± 1.47	3.12 ± 1.45^*^	3.22 ± 1.46^^*^, †^	2.99 ± 1.47	$\eta_{p}^{2}$ =	0.013	0.016	0.002

*PA, physical activity; Avg, average*.

§ *Different from females (*p* < 0.001);*

# *Different from females (*p* = 0.017);*

* *Different from Primary (*p* < 0.001);*

† *Different from Secondary (*p* < 0.001). Bold values are the main effects of the dependent variables*.

## Discussion

The aim of this study was to identify sex and educational stage differences in the barriers to PA in children and adolescents from 5th grade of primary school to 2nd grade of college. The major finding was, on one hand, that female children and adolescents report higher disliking and time barriers to PA than their male counterparts in the entire educational pathway.

Our results showed no effect of sex on environmental and safety barriers, which contrasts with a previous study indicating greater environmental barriers for girls (Jongenelis et al., 2018). Greater disliking and time barriers observed in females could be between the underlying reasons for the higher levels of MVPA observed in boys at all educational stages in comparison to girls, which is in accordance with the existing literature (Madsen et al., 2009; Fernández et al., 2017; Guthold et al., 2020; Rosselli et al., 2020). Previous studies have also observed that girls report more barriers to PA than boys (Jodkowska et al., 2015; Rosselli et al., 2020; Lazarowicz et al., 2021). Specifically, within disliking reasons, the lack of skills is one of the major barriers reported by girls (Jodkowska et al., 2015). Low perceive competence is even more exacerbated in overweight girls and may be affected by the pressure to perform well in team sports, and altogether with fear of criticism and embarrassment, especially in the presence of males, hold a negative attitude toward exercise and act as a barrier for girls to participate and attempt new activities (Jodkowska et al., 2015; Corr et al., 2019; Cowley et al., 2021). Another major reason within disliking barriers highly stated in girls is tiredness or lack of energy and willpower (Fernández et al., 2017; Rosselli et al., 2020). In fact, a previous study indicated that the greater the distance from classroom to schoolyard facilities the greater recess in schoolyard PA, especially in older girls (Pawlowski et al., 2019). Both, lack of skills and tiredness are items included in the disliking barriers group provided by the questionnaire used in our study. However, we have not evaluated the weight of the different items in the entire disliking barriers group, which should be considered in future studies. In fact, tiredness together with body image reasons have been considered the most relevant perceived barriers to perform PA, especially in females (Fernández et al., 2017). Previous research has suggested that girls feel a pressure to look good when exercising and reluctance to sweat and wear not-fitting uniforms, altogether results in serious barriers to participation (Rosselli et al., 2020; Cowley et al., 2021; Duffey et al., 2021). In this regard, despite being previously validated, our questionnaire did not include body image items, which may be considered as a limitation and should also be addressed in future studies. Interestingly, in our Spanish sample, disliking barriers resulted less rated than time barriers which may be related to the increase in school workload or even in home responsibilities (Corr et al., 2019; Duffey et al., 2021). This seems to especially affect females, since in the same way as it occurs with disliking reasons, female students in our study reported greater time barriers than their male counterparts, which agrees with other studies (Rosselli et al., 2020; Lazarowicz et al., 2021). A reason that literature states is the change in leisure activities, especially girl's desire to do different things like shopping or hanging out with friends instead of being physically active (Corr et al., 2019; Rosselli et al., 2020). Another reason suggested is the remaining socio-cultural pattern of increased home duties and household work in girls in comparison to boys (Lazarowicz et al., 2021). There were also studies showing no sex differences in time barriers (Fernández et al., 2017) or even no sex differences in the perception of any barriers (Gunnell et al., 2015). However, it is important to highlight that all these findings are not entirely comparable with ours since neither the educational stages nor the range of student ages evaluated nor the perceived barriers questionnaire used were the same.

On the other hand, in terms of differences among educational stages, primary school turned out to be the most active educational stage in comparison to the other two stages, which is in accordance with previous studies (Dumith et al., 2011; Jago et al., 2012; Van Hecke et al., 2016), and likewise, in disliking, safety and time barriers there were differences from primary school to the other two educational stages. Both time and disliking barriers were more prevalent among secondary and college students than among primary school students. The lack of time has been shown as a recurrent barrier for both girls and boys, especially in secondary and college stages (Calogiuri and Chroni, 2014; Jodkowska et al., 2017; Divyasree et al., 2018) being the increasing workload and academic demands suggested as the main reasons (Harvey et al., 2018) while greater disliking barriers from primary school onwards have been observed by previous authors (Jodkowska et al., 2015; Payán et al., 2019) and could be due to a decrease in motivation to PA in the transition from childhood to adolescence (Martins et al., 2015) and a preference for other non-physically active but more social activities (Corr et al., 2019; Rosselli et al., 2020). In contrast, the safety barrier resulted higher in primary school than in later stages. These results may be easily explained by the gradual disappearance of misgivings about outdoor PA as children grow older and their families give them greater autonomy and greater levels of responsibility for moving around on their own and managing their time, although authors such as Marconnot et al. (2019) and Vasquez et al. (2021) continue to mention this barrier among older adolescents, with lesser extent than among primary school students though. Finally, the two barriers showing interaction between sex and educational stage are the lack of time and dislike of PA practice. Both barriers are perceived more strongly by girls than boys, although both sexes mention these barriers more frequently in adolescence than in childhood, which is in accordance with previous studies (Jodkowska et al., 2017; Divyasree et al., 2018). In the college period, only girls reported further increased barriers of time and dislike for PA practice in comparison to secondary. In this regard, the above stated ideas regarding greater tiredness in older girls and their interest in other activities different from PA for their leisure time could explain our result (Jodkowska et al., 2015; Corr et al., 2019; Rosselli et al., 2020; Cowley et al., 2021; Duffey et al., 2021; Lazarowicz et al., 2021).

Finally, some limitations should be stated. First of all, the use of subjective self-reported instruments may elicit errors related to respondent recall or desirability bias. Moreover, the use of different questionnaires in literature (Niñerola et al., 2006; Rosselli et al., 2020) despite being all validated and reliable, result in different items and barrier groups evaluated, thus impairing an accurate comparison of findings. Indeed, as previously mentioned, our study did not include body centered issues (Fernández et al., 2017), but either items related to peers, family and friends support (Dishman et al., 2017; Corr et al., 2019; Mehtälä et al., 2020; Cowley et al., 2021) or items related to screen-based recreation very present in adolescents' life nowadays (Jongenelis et al., 2018; Mehtälä et al., 2020). Therefore, to elaborate a more comprehensive questionnaire, more connected with current adolescent population should be interesting for future studies. Additionally, differences in educational systems worldwide in terms of stages and grades should be cautiously considered when different countries are compared to determine if differences in barriers to PA (e.g., time barriers), are more depending on higher workload associated to the educational stage or they are more related to psychosocial processes associated to age development.

In terms of applicability, the results obtained in the present study should encourage the reflection on the policies that should be carried out from health and educational spheres, in order to reduce the time and displeasure-related barriers to PA, especially in adolescent girls. Strategies should increase the opportunities to practice sport regardless of educational stage and sex, taking into account the specific needs and demands of each population group in order to provide an adapted response to them. Close collaboration between the education administration and physical activity promoters is needed for proper time management, given the proven evidence of the benefits associated with daily physical activity.

## Conclusion

Throughout the different educational stages of the Spanish Educational System, the main sex differences in barriers to PA are the lack of time and dislike of PA which female students rated higher than their male counterparts. In addition, a gradual decrease or abandonment of PA is observed from primary school onwards, likewise more accentuated in females. These findings may suggest that Spanish PA promotion strategies should carefully consider girls and the step into secondary school.

## Data Availability Statement

The raw data supporting the conclusions of this article will be made available by the authors, without undue reservation.

## Ethics Statement

The studies involving human participants were reviewed and approved by Comité de Ética de la Universidad Rey Juan Carlos. Written informed consent to participate in this study was provided by the participants' legal guardian/next of kin.

## Author Contributions

JD-D-L-M: conception and design of the study. JD-D-L-M, DB-G, and AS-A: data collection. NR-P: data analyses and interpretation. DB-G and NR-P wrote the initial draft. All authors critically reviewed the content and approved the final version.

## Conflict of Interest

The authors declare that the research was conducted in the absence of any commercial or financial relationships that could be construed as a potential conflict of interest.

## Publisher's Note

All claims expressed in this article are solely those of the authors and do not necessarily represent those of their affiliated organizations, or those of the publisher, the editors and the reviewers. Any product that may be evaluated in this article, or claim that may be made by its manufacturer, is not guaranteed or endorsed by the publisher.
